# Advanced chronic kidney disease increases complications in anterior cervical discectomies with fusions: An analysis of 75,508 patients

**DOI:** 10.1016/j.xnsj.2024.100331

**Published:** 2024-05-24

**Authors:** Christopher G. Hendrix, Haseeb E. Goheer, Alden H. Newcomb, Jonathan J. Carmouche

**Affiliations:** a Department of Orthopaedic Surgery, Institute for Orthopaedics and Neurosciences, Carilion Clinic, 2331 Franklin Road Southwest, Roanoke, VA 24014, United States; b Virginia Tech Carilion School of Medicine, 2 Riverside Circle, Roanoke, VA 24016, United States

**Keywords:** Anterior cervical discectomy and fusion, NSQIP, Kidney disease, Complications, Cervical spine, ACDF, CKD

## Abstract

**Background:**

Although anterior cervical discectomy and fusion (ACDF) procedures for cervical spine disease have been increasing amid a growing population of patients with kidney dysfunction, there is a scarcity of literature focusing on kidney dysfunction as a risk-factor for post-operative ACDF complications. The purpose is to evaluate the differential impact of kidney dysfunction on perioperative outcomes including surgical and medical complications, extended length of hospital stay (LOS), and death within 30 days following ACDF.

**Patient Sample:**

This was a retrospective cohort study of prospectively collected data using the American College of Surgeons National Surgical Quality Improvement Program database to identify patients who had undergone an elective ACDF procedure between 2011-2021 using Current Procedural Terminology code 22551. Patients were categorized into five cohorts based on eGFR according to the “Kidney Disease: Improving Global Outcomes” Classification: values of: ≥ 90(reference cohort), 60-89 (G2), 30-59 (G3), 15-29 (G4), and <15 (G5). One-way ANOVA for continuous variables and chi-square tests for categorical variables were used to identify differences in perioperative variables between the five groups. Multivariable logistic regression analysis assessed the effect of kidney dysfunction on post-operative surgical outcomes. Significance was defined as p<.05.

**Results:**

About 75,508 ACDF patients were included, of who 57,480 were G1, 15,186 were G2, 2,192 were G3, 312 were G4, and 338 were G5. G4 and G5 independently increased the risk of medical complications (OR: 1.893, 95% CI [1.296-2.705]; OR: 2.241, 95% CI [1.222-3.964]) and blood transfusion. Only G5 independently increased the risk for extended LOS (OR: 2.410, 95% CI [1.281-4.371], p=.005).

**Conclusion:**

High grade CKD is an independent risk factor for medical complications, extended hospital LOS, and blood transfusions following ACDF, underscoring the importance of risk stratification to optimize perioperative management and reduce the burden of complications and healthcare costs. Conversely, low grade CKD does not increase the risk of complications in ACDF.

## Introduction

Anterior cervical discectomy and fusion (ACDF), one of the most performed elective procedures in the United States, has seen a steady increase in utilization over the years [[1], [2], [3], [4]]. Regarded as the preferred approach for treating cervical radiculopathy and myelopathy, despite the potential risks, ACDF is a procedure with a low complication rate.

The anterior approach's proximity to vital structures such as neuro-vasculature, fascial planes, and the aerodigestive tract introduces a diverse array of potential complications. Specific complications associated with ACDF include dysphagia, recurrent laryngeal nerve palsy, adjacent segment disease, pseudoarthrosis, graft or hardware failure, hematoma, esophageal perforation, and spinal cord injury [5]. Complications able to be evaluated in this study such as bleeding, prolonged hospital stay, and death are also important risks in ACDF as with any surgery. Excessive bleeding in ACDF surgeries can obscure the surgeon's view, heightening the risk of inadvertent injury to nearby structures, and potentially necessitating blood transfusions with associated risks of transfusion reactions and disease transmission. Prolonged hospital stays post-ACDF increase financial burdens of both the patient and healthcare system, raise the risk of infections and complications like thrombosis and pneumonia, and may disrupt patients' lives, causing emotional distress and delaying their return to normal activities. Though death is increasingly rare in ACDF surgeries [5], parsing out significant factors associated with ACDF leading to death is gravely important.

With the surge in outpatient ACDF procedures and the growing incidence of cervical disc disease among older individuals, ACDF's popularity is on the rise [4,6]. Concurrently, chronic kidney disease (CKD) is also escalating due to factors like diabetes, hypertension, and an aging populace. From 2005 to 2020, CKD prevalence in US adults increased from 12.5% to 14%, affecting approximately 1 in 7 individuals [7]. However, this figure likely underrepresents reality, given that most CKD cases are asymptomatic [8]. For instance, up to 30% of cardiac surgery patients are estimated to have preexisting renal conditions [9].Consequently, the number of ACDFs performed on patients with kidney dysfunction is significant and will likely rise.

Patients with CKD have traditionally experienced worse perioperative results, such as postoperative acute kidney injury, major adverse cardiac events, and higher mortality rates [[10], [11], [12], [13]]. They are also at greater risk of hemodynamic instability and drug-related complications [10,11]. CKD is a notable predictor of mortality [12,14] in ACDF procedures and is linked to higher rates of unplanned readmissions [15].

In the past decade, there has been a lack of data on the connection between CKD and ACDF, especially regarding perioperative outcomes and the impact of worsening kidney function. To address this gap, we intend to utilize the American College of Surgeons National Surgical Quality Improvement Program (ACS-NSQIP) database to assess how increasing kidney dysfunction affects short-term morbidity and mortality in ACDF patients.

## Materials and methods

### Study design and data sources

This is a retrospective, multi-center cohort study of patient data from the ACS-NSQIP database. ACS-NSQIP is a nationally validated, outcome-based database with data collected from over 600 hospitals across the United States by certified healthcare workers. The database undergoes regular quality assurance with an inter-reliability disagreement rate of less than 2% [16]. The database captures perioperative variables including demographic characteristics and comorbidities, intraoperative variables, and 30-day outcomes.

### Study population and outcomes of interest

Current Procedural Terminology Code (CPT) 22551 was queried to identify 85,758 patients who underwent ACDF from 2011 to 2021. The CPT code 22552 was used to identify procedures involving multiple operative levels (i.e., 2-level ACDF, 3-level ACDF). Patients with a missing preoperative serum creatinine value and/or sex were excluded. The exclusions resulted in the inclusion of a total of 75,508 patients (88.1%) cases for statistical analysis. The following Mayo Quadratic equation was chosen to calculate the estimated glomerular filtration rate based on its accuracy in previous studies [17]: eGFR = exp [1.911 + 5.249/sCr − 2.114/sCr2 − 0.00686  ×  Age − (0.205 if female)], if sCr < 0.8 mg/dL then sCr = 0.8. The study population was then divided into five cohorts based on CKD-staging guidelines using eGFR, G1 (≥90 mL/min/1.73m²), G2 (≥60 and <90 mL/min/1.73m²), G3 (≥30 and <60 mL/min/1.73m²), G4 (≥15 and <30 mL/min/1.73m²), and G5 (<15 mL/min/1.73m²) [18]. G1 is the reference cohort for the study.

Patient and clinical characteristics including age, body mass index (BMI), sex, race, admission status, smoking history, chronic obstructive pulmonary disease (COPD), congestive heart failure (CHF), history of cancer, bleeding disorders, steroid use, dialysis, elective surgery, ventilation, transfusion, pre-operative sepsis, hypertension requiring medication, diabetes mellitus, functional status, and American Society of Anesthesiologists (ASA) class status were collected and available in the ACS-NSQIP for all years of interest for the present study. The variables clinically relevant to the outcome measures of the presented study were selected from the database. Due to the presence of these characteristics across all years selected, we were able to make comparisons to previously reported odds ratios.

Primary outcomes were 30-day surgical complications, medical complications, bleeding requiring transfusions, extended length of hospital stay (LOS), and death. Surgical complications include superficial incisional surgical site infection (SSI), deep incisional SSI, organ space SSI, and wound dehiscence. Medical complications include pneumonia, unplanned intubation, pulmonary embolism, failure to wean from a ventilator after 48 hours, acute renal failure, urinary tract infection (UTI), cerebral vascular accidental (CVA)/stroke with neurological deficit, cardiac arrest requiring cardiopulmonary resuscitation (CPR), myocardial infarction (MI), blood transfusions, deep vein thrombosis (DVT), sepsis, septic shock, extended LOS, and death. Extended LOS is defined as a total LOS greater than 2 times the standard deviation above the mean of total LOS for all patients analyzed in the study.

### Statistical analysis

One-way analysis of variance (ANOVA) for continuous variables and chi-square tests for categorical variables were used to assess differences in pre- and perioperative variables across the G1, G2, G3, G4, and G5 cohorts. Multivariate logistic regression analysis was used to determine independent associations for medical complications, surgical complications, blood transfusions, extended LOS, and death within 30 days of surgery. We utilized a liberal criterion of p≤.20 to include preoperative variables to minimize bias in the multivariate logistic regression as previously advocated [19], [20]. The significant characteristics controlled for in the multivariate analyses included the following: age, BMI, sex, race, admission status, smoking history, COPD, CHF, cancer history, bleeding disorders, steroid use, dialysis, elective surgery, ventilation, transfusion, sepsis, hypertension, diabetes, functional status, ASA class, and CKD group. Significance was defined as p<.05. R was used to conduct all statistical analyses. ACS-NSQIP and the hospitals participating in the ACS-NSQIP are the source of the data used herein; they have not verified and are not responsible for the statistical validity of the data analysis or the conclusions derived by the authors.

## Results

Between 2011 and 2021, 75,508 ACDF procedures were identified, of which 57,480 (76.1%) were G1, 15,186 (20.1%) were G2, 2,192 (2.9%) were G3, 312 (0.4%) were G4, and 338 (0.4%) were G5. Baseline demographics and comorbidities were compared among the groups (Table 1). Statistically significant differences in demographic factors (e.g., age, BMI, sex, and race), admission status, and comorbidities (e.g., smoking, COPD, CHF, cancer, bleeding disorders, steroid use, dialysis, elective surgery, ventilation, transfusion, sepsis, hypertension, diabetes, functional status, and ASA class) were identified across the 5 cohorts. Patients with higher grade CKD had increased rates of pre-operative bleeding disorders (1.9%, 3.4%, 6.4%, 6.5% vs. 1.1%; p<.001).

**Table 1:** Demographics variables and comorbidities in patients undergoing ACDF (N=75,508).

	Normal n=57,480	G2 n=15,186	G3 n=2,192	G4 n=312	G5 n=338	p
Age (SD)	52.80 (10.44)	65.55 (9.88)	67.79 (9.69)	67.04 (9.88)	60.33 (10.54)	<.001
BMI (SD)	30.57 (6.86)	30.62 (6.50)	31.30 (6.61)	31.77 (7.28)	29.77 (6.84)	<.001
Sex						<.001
Female	47.9%	59.4%	38.9%	36.2%	61.0%	
Male	52.1%	40.6%	61.1%	63.8%	39.0%	
Race						<.001
White	78.5%	78.1%	72.9%	66.0%	51.5%	
Black	10.3%	13.6%	19.6%	22.8%	32.5%	
Asian	1.9%	1.8%	1.7%	1.9%	3.3%	
American Indian or Alaskan Native	0.7%	0.4%	0.2%	0.6%	1.8%	
Native Hawaiian or Pacific Islander	0.3%	0.3%	0.3%	0.3%	0.9%	
Unknown	8.3%	5.8%	5.3%	8.3%	10.1%	
Admission Status						<.001
Inpatient	63.7%	67.3%	70.1%	79.8%	86.7%	
Outpatient	36.3%	32.7%	29.9%	20.2%	13.3%	
Smoking History	28.9%	16.2%	15.8%	15.1%	19.8%	<.001
Chronic Obstructive Pulmonary Disease	4.1%	6.6%	9.1%	10.6%	10.1%	<.001
Congestive Heart Failure	0.3%	0.8%	2.7%	5.5%	7.4%	<.001
Cancer History	0.2%	0.3%	0.6%	0.6%	0.6%	<.001
Bleeding Disorders	1.1%	1.9%	3.4%	6.4%	6.5%	<.001
Steroid Use	3.4%	4.9%	6.8%	9.6%	6.2%	<.001
Dialysis	0.0%	0.1%	0.2%	6.4%	69.0%	<.001
Elective Surgery	93.6%	94.3%	92.1%	82.7%	66.2%	<.001
Ventilation	0.1%	0.1%	0.2%	1.3%	1.5%	<.001
Transfusion	0.1%	0.1%	0.2%	1.3%	4.4%	<.001
Sepsis	0.8%	0.9%	1.9%	4.5%	9.5%	<.001
Hypertension	42.5%	66.8%	85.1%	88.1%	78.7%	<.001
Diabetes	14.5%	24.1%	41.8%	54.5%	40.2%	<.001
Multiple Levels	47.6%	51.2%	50.9%	46.8%	42.6%	<.001
Functional Status						<.001
Independent	98.0%	96.6%	96.0%	88.8%	81.7%	
Partially Dependent	1.4%	2.6%	3.3%	9.0%	16.0%	
Totally Dependent	0.2%	0.3%	0.2%	1.3%	2.1%	
Unknown	0.4%	0.6%	0.5%	1.0%	0.3%	
ASA Class						<.001
I: No Disturbance	3.3%	0.9%	0.2%	0.0%	0.0%	
II: Mild Disturbance	53.0%	37.4%	19.4%	6.1%	10.7%	
III: Severe Disturbance	42.0%	58.2%	71.9%	74.4%	47.6%	
IV: Life Threatening	1.8%	3.5%	8.5%	18.9%	41.7%	
V: Moribund	0.0%	0.0%	0.0%	0.6%	0.0%	

ASA, American Society of Anesthesiologists.

The rates of postoperative complications are listed in Table 2. About 2,664 patients had at least one surgical and/or medical complication. Of these patients with at least one complication, 991 (37.2%) had pre-operative kidney dysfunction. There was no statistical difference in overall surgical complications between the 5 groups of kidney dysfunction. The rates of medical complications, UTI, blood transfusion, MI, sepsis, and postoperative death within 30-days increased from G1 through G5. The G2, G3, G4, and G5 groups experienced longer LOS at 2.1, 2.2, 4.8, and 6.2 days, compared with 1.8 days for the G1 group (p<.001).

**Table 2:** Univariate analysis of thirty-day post-operative complications after anterior cervical discectomy and fusion by chronic kidney disease cohort.

	Normal n=57,480	G2 n=15,186	G3 n=2,192	G4 n=312	G5 n=338	p
**Surgical Complications**	0.7%	0.7%	0.7%	0.6%	1.8%	.188
Superficial Surgical Site Infection (SSI)	0.3%	0.2%	0.4%	0.0%	0.3%	.198
Deep SSI	0.1%	0.2%	0.2%	0.0%	0.3%	.480
Organ SSI	0.2%	0.2%	0.1%	0.6%	0.9%	.006
Wound Disruption	0.1%	0.1%	0.1%	0.0%	0.3%	.417
Medical Complications	2.4%	4.2%	6.6%	15.7%	21.3%	<.001
Pneumonia	0.7%	1.3%	1.8%	5.8%	5.0%	<.001
Unplanned Intubation	0.5%	0.9%	1.0%	5.1%	4.7%	<.001
Pulmonary Embolism	0.2%	0.3%	0.4%	0.3%	0.6%	.016
Ventilator > 48 hours	0.4%	0.7%	1.3%	4.2%	3.9%	<.001
Acute Renal Failure	0.0%	0.1%	0.3%	1.0%	0.6%	<.001
Urinary Tract Infection	0.5%	1.0%	1.7%	2.6%	3.0%	<.001
CVA/Stroke with Neuro Deficit	0.1%	0.1%	0.3%	0.0%	0.6%	<.001
Cardiac Arrest Requiring CPR	0.1%	0.3%	0.5%	1.6%	2.4%	<.001
Myocardial Infarction	0.1%	0.3%	0.4%	1.3%	1.5%	<.001
Blood Transfusions	0.3%	0.8%	1.2%	4.5%	8.0%	<.001
Deep Vein Thrombosis	0.3%	0.4%	0.7%	1.5%	2.1%	<.001
Sepsis	0.3%	0.4%	0.8%	2.6%	3.9%	<.001
Septic Shock	0.1%	0.2%	0.5%	1.9%	1.2%	<.001
Extended LOS	2.0%	3.0%	4.9%	11.5%	27.2%	<.001
**Death**	0.2%	0.5%	1.2%	4.5%	4.7%	<.001

ACDF, anterior cervical discectomy and fusion; CPR, cardiopulmonary resuscitation; CVA, cerebral vascular accident; LOS, length of stay.

Multivariate logistic regression was performed to determine the differential effect of kidney dysfunction status compared to the normal G1 group on surgical complications, medical complications, extended LOS, blood transfusions, and death while adjusting for the greater comorbidity burden (Table 3). G4 and G5 independently increased the risk of medical complications (OR: 1.893, 95% CI [1.296-2.705]; OR: 2.241, 95% CI [1.222-3.964]). Only G5 independently increased the risk for extended LOS (OR: 2.410, 95% CI [1.281-4.371], p=.005) while G4 increased the risk for death (OR: 2.073, 95% CI [0.971-4.046], p=.044). G3, G4, and G5 independently increased the risk of blood transfusions (OR: 1.879, 95% CI [1.142-2.984]; OR: 3.957, 95% CI [1.912-7.575]; OR: 6.755, 95% CI [2.343-10.705]) needed following an ACDF. Table 4 includes an expanded multivariate logistic regression with no variable reaching significance.

**Table 3:** Multivariate adjusted 30-day ACDF postoperative outcomes among CKD groups compared to non-CKD.

Overall Cohort	G2 (n=15,186)			p	G3 (n=2,192)			p
	Adjusted Odds Ratio	95% CI			Adjusted Odds Ratio	95% CI		
		Lower	Upper			Lower	Upper	
Surgical Complications	0.950	0.737	1.215	.688	0.775	0.431	1.292	.359
Medical Complications	1.031	0.917	1.158	.606	1.148	0.935	1.400	.180
Extended Hospital LOS	0.892	0.777	1.020	.010	0.908	0.715	1.144	.422
Blood Transfusions	1.297	0.959	1.744	.088	1.879	1.142	2.984	**.010**
Death	0.776	0.546	1.098	.157	1.167	0.718	1.839	.516

Overall Cohort	G4 (n= 312)			p	G5 (n= 338)			p
	Adjusted Odds Ratio	95% CI			Adjusted Odds Ratio	95% CI		
		Lower	Upper			Lower	Upper	

Surgical Complications	0.499	0.081	1.627	.339	1.502	0.265	5.507	.604
Medical Complications	1.893	1.296	2.705	**.001**	2.241	1.222	3.964	**.007**
Extended Hospital LOS	1.105	0.703	1.675	.661	2.410	1.281	4.371	**.005**
Blood Transfusions	3.957	1.912	7.575	**<.001**	6.755	2.343	10.705	**<.001**
Death	2.073	0.971	4.046	.044	1.471	0.397	4.943	.554

ACDF, anterior discectomy and fusion; CI, confidence interval; LOS, length of stay.

**Table 4:** Multivariate adjusted 30-day ACDF post-operative outcomes among CKD groups compared to non-CKD.

Overall Cohort	G2 (n=15,186)			p	G3 (n=2,192)			p
	Adjusted Odds Ratio	95% CI			Adjusted Odds Ratio	95% CI		
		Lower	Upper			Lower	Upper	
Pneumonia	0.992	0.807	1.215	.935	0.833	0.572	1.185	.326
Unplanned Intubation	0.988	0.774	1.254	.919	0.679	0.414	1.061	.105
Pulmonary Embolism	1.087	0.744	1.572	.658	0.854	0.369	1.722	.683
Ventilator > 48 hours	0.898	0.669	1.175	.416	1.001	0.625	1.543	.997
Cardiac Arrest Requiring CPR	0.938	0.594	1.461	.779	1.032	0.492	1.986	.929
Myocardial Infarction	1.074	0.677	1.688	.758	0.969	0.43	1.959	.934
Deep Vein Thrombosis	0.936	0.658	1.315	.707	1.273	0.692	2.188	.408
Sepsis	1.001	0.704	1.407	.993	1.242	0.692	2.102	.440
Septic Shock	1.304	0.769	2.181	.317	1.323	0.571	2.796	.486

Overall Cohort	G4 (n=312)			P	G5 (n=338)			p
	Adjusted Odds Ratio	95% CI			Adjusted Odds Ratio	95% CI		
		Lower	Upper			Lower	Upper	

Pneumonia	1.429	0.77	2.482	.229	1.293	0.409	3.571	.655
Unplanned Intubation	0.679	0.414	1.061	.028	0.695	0.212	2.201	.547
Pulmonary Embolism	0.441	0.224	2.2268	.447	0.851	0.376	7.858	.913
Ventilator > 48 hours	1.386	0.606	2.822	.401	0.549	0.132	2.188	.410
Cardiac Arrest Requiring CPR	1.602	0.428	4.439	.421	1.006	0.175	5.339	.994
Myocardial Infarction	1.729	0.414	5.122	.382	2.15	0.331	10.541	.393
Deep Vein Thrombosis	2.095	0.687	5.062	.139	1.993	0.359	8.290	.401
Sepsis	1.927	0.754	4.275	.134	1.352	2.929	5.325	.689
Septic Shock	2.601	0.78	7.104	.085	1.415	1.253	1.104	.774

## Discussion

In the current study, nearly one-quarter (23.87%) of the over 75,000 patients were found to have pre-operative kidney dysfunction. This finding reinforces that the overall prevalence of CKD among Americans is likely underestimated due to the initial asymptomatic nature of the disease and align with a study by Warth et al. [20] finding that 26%-27% of patients undergoing total joint arthroplasty have CKD, and with the findings of Rosner et al. [9], who reported that up to 30% of patients undergoing cardiovascular surgery have preoperative kidney dysfunction. Furthermore, it is important to highlight that in this analysis, among the 3.53% of patients who experienced a complication, more than one-third (37.2%) of them had pre-operative kidney dysfunction.

Patients with pre-existing kidney dysfunction showed a higher prevalence of comorbidities, along with older age, higher BMI, ASA class, and lower functional status on average. Patients with pre-operative kidney dysfunction did not have an increased risk of surgical complications. These patients demonstrated an elevated risk of medical complications and mortality when compared to the normal kidney function group in the unadjusted analysis, with a progressive increase in mortality rate as kidney function declined. We did not find an independent association between groups G2-G5 and increased 30-day mortality after adjusting for confounders. Independent associations were found between groups G3-G5 and increased blood transfusions, additionally, groups G4 and G5 independently increased the risk of medical complications, while group G5 exhibited an increased risk of extended LOS.

CKD is a significant risk factor in developing perioperative complications in other spine surgeries [21], with CKD patients having increased risk of ICU transfer, delirium, UTI, DVT, and 30-day readmissions [21] in patients undergoing lumbar decompression and fusion. CKD patients after elective joint arthroplasties reportedly have increased time to discharge, sustain more pulmonary, infectious, cardiovascular, renal, and neurological complications, and has been found to be an independent risk factor for prolonged LOS, as well as postoperative acute on chronic kidney injury in patients after a total hip arthroplasty [[22], [23], [24]]. CKD patients have also been found to have increased mortality in spine surgery [[10], [11], [12]]. Hanci et al. [25] found that the rate of acute kidney injury after major orthopedic surgery to be 7.1%, and 11% after total joint arthroplasties alone, and a rate as high as 17% was found in a meta-analysis by Li et al. [26] after hip fracture surgery.

CKD exerts a tremendous impact on healthcare costs, representing a disproportionate 20% of the health budget and affecting 1 out of every 7 Americans [27]. As renal function declines, individual patient expenses rise, with estimated 4-month CKD management costs ranging from $7,725 for lower stage disease to $11,879 for higher stages (without renal replacement therapy). Notably, costs escalated significantly to $87,538 and $124,271 for patients on dialysis and for kidney transplantation, respectively [28]. With valued-based care remaining a national priority, and our findings that perioperative outcomes in ACDF increase significantly above a disease severity threshold, it warrants further discussion on the impact it may have on the risk-stratification and adjustment process as well as the cost-saving implications. It is important to acknowledge that with low grade CKD in this study, there was no increased risk of complications following ACDF within 30-days postoperatively. Though renal status should be optimized as much as possible for surgery, surgeons can safely perform ACDF in low grade CKD patients.

Acknowledgment of several limitations is warranted in this study. The retrospective design prevents randomization and control of confounding variables, such as variations in postoperative wound care protocols. The analysis is limited to the 30-day postoperative period, which may result in the exclusion of unreported complications and functional outcomes beyond this timeframe, such as pseudoarthrosis or hardware failure. These complications are crucial to consider when assessing the overall success and potential risks of ACDF procedures. Results of database studies are descriptive and do not offer solutions beyond the recommendations given in this study. To gain a more comprehensive understanding of outcomes, future studies should aim to utilize databases with a longer postoperative window to identify potential complications occurring at later stages or perform prospective studies. Although demographic data were included, factors such as socioeconomic status and insurance were not accounted for. While a single versus multilevel analysis was completed, missing CPT codes in the database may have biased the identification of this variable.

## Conclusions

This study highlights the significance of pre-operative kidney dysfunction in ACDF patients, underscoring the importance of risk stratification to optimize perioperative management and reduce the burden of complications and healthcare costs. The elevated risk of medical complications in patients with high grade CKD, blood transfusions, extended LOS, and mortality necessitates careful consideration of preoperative kidney function assessment. Conversely, low grade CKD does not increase the risk of complications within the 30-day postoperative window following ACDF. This study raises important questions about the true risk-to-benefit ratio for patients in the G4, and especially G5 groups when considering elective ACDF.

## IRB statement

Institutional review board approval was not required since the present study utilized de-identified data derived from a national healthcare dataset.

## Declaration of competing interest

The authors declare that they have no known competing personal or financial interests that could have appeared to influence this study.
